# ‘I suppose language is important’: investigating news media and third sector views on food poverty

**DOI:** 10.1093/heapro/daaf073

**Published:** 2025-06-10

**Authors:** Claire Kerins, Sinéad Furey, Páraic Kerrigan, Aodheen McCartan, Colette Kelly, Eimer Brown, Nisha Neelakant, Elena Vaughan

**Affiliations:** School of Health Sciences, Health Promotion Research Centre, University of Galway, University Road, Galway H91 TK33, Ireland; School of Nursing & Midwifery, Centre for Health Research Methodology, University of Galway, University Road, Galway H91 TK33, Ireland; Department of Hospitality, Tourism and Events Management, Ulster University Business School, Ulster University, Cromore Road, Coleraine, Co. Londonderry BT52 1SA, United Kingdom; School of Information and Communication Studies, University College Dublin, Belfield, Dublin 4 D04 V1W8, Ireland; School of Communication and Media, Ulster University, York Street, Belfast, Co. Antrim BT15 1ED, United Kingdom; School of Health Sciences, Health Promotion Research Centre, University of Galway, University Road, Galway H91 TK33, Ireland; School of Health Sciences, Health Promotion Research Centre, University of Galway, University Road, Galway H91 TK33, Ireland; School of Medicine, University of Galway, University Road, Galway H91 TK33, Ireland; School of Health Sciences, Health Promotion Research Centre, University of Galway, University Road, Galway H91 TK33, Ireland

**Keywords:** food poverty, food insecurity, framing theory, news media, third sector, food charity, qualitative methods

## Abstract

News media coverage can shape public and political perceptions of food poverty, yet little is known about how media professionals and third sector organizations understand and communicate this issue. This study aimed to explore how food poverty is understood and communicated by news media professionals and third sector representatives on the island of Ireland. Semi-structured interviews were conducted with news media professionals (*n* = 16) and third sector representatives (*n* = 14) from the Republic of Ireland and Northern Ireland. A combination of deductive coding to Entman's framing theory and inductive thematic analysis was used to identify perspectives on food poverty and its media representation. The findings revealed distinct perspectives between groups regarding causes and solutions. Third sector representatives emphasized structural drivers and advocated policy solutions, while media professionals’ views were more mixed, with some emphasizing individual responsibility. Media professionals reported preferring case studies featuring families and single mothers, while third sector representatives expressed concerns about their role as gatekeepers. The study identified a mutual reliance between news media and third sector organizations in the processes of securing case studies and sharing information. Both groups reported challenges with resource constraints, ethical considerations, and communicating complex issues. These findings reveal how institutional arrangements between media and third sector organizations may reinforce individualistic narratives of food poverty rather than structural causes. The results suggest a need for strategic approaches including evidence-based reporting guidelines and improved access to independent data sources and expertise to support more effective communication of structural drivers and the need for policy solutions.

Contribution to Health Promotion

- This study provides novel insights into how news media professionals and third sector organizations understand and communicate about food poverty.
- Findings suggest the way news media and third sector organizations work together may unintentionally emphasize individual responsibility, despite evidence supporting the need to address the structural causes of food poverty.
- Results highlight the need for strategies, such as media guidelines and access to reliable data and expert sources, to better communicate structural causes and policy solutions.
- Health promotion practitioners can collaborate with media and third sector organizations to develop communication strategies that reframe food poverty as a human rights issue.

## INTRODUCTION

Food poverty and insecurity have emerged as growing societal and public health problems across economically developed countries (Gundersen and Ziliak 2018). While there are differences in definition and dimensions, food poverty and food insecurity are considered interrelated concepts, both addressing inadequate access to sufficient, safe, and nutritious food (O’Connor *et al.* 2016). Food poverty is defined as ‘the insufficient economic access to an adequate quantity and quality of food to maintain a nutritionally satisfactory and socially acceptable diet’ (O’Connor *et al.* 2016, p. 429), while food insecurity refers to ‘the inability to consume an adequate quality or sufficient quantity of food in socially acceptable ways, or the uncertainty that one will be able to do so’ (Dowler and O’Connor 2012 , p. 45).

While food insecurity is most commonly associated with the developing world (Middleton *et al.* 2018), household food insecurity in high-income countries is a longstanding, but largely overlooked, serious public health concern (Loopstra 2018). There is a paucity of quantitative evidence on trends in the prevalence of food insecurity in rich countries (Davis and Geiger 2017). Studies indicate that the prevalence of household food insecurity in some developed countries ranges from 8% to 20% of the population (Tarasuk and Mitchell 2020, FAO, IFAD, UNICEF, WFP, and WHO 2023). In the Republic of Ireland (ROI), ∼9% of the population is estimated to be in food poverty (Food Poverty Working Group 2022). In Northern Ireland (NI), recent data from the Food Standards Agency (2024) indicate that 27% (*n* = 418) of respondents were classified as food insecure (13% low food security, 14% very low security) (Food Standards Agency 2024). The Family Resources Survey 2022/23 similarly identified 4% of households experiencing very low food security (Northern Ireland Statistics and Research Agency 2024).

### Health impacts of food poverty and insecurity

The consequences of food poverty and insecurity are wide-ranging and severe for individuals and families. Meta-analyses have shown that food insecurity in adults is associated with micronutrient deficiency and increased risk of anaemia (Lopes *et al.* 2023), chronic conditions such as obesity, diabetes, and heart disease and multimorbidity (Kantilafti *et al.* 2023), as well as higher rates of depression and anxiety (Pourmotabbed *et al.* 2020). Systematic reviews have demonstrated that children in food insecure households have an increased risk of stunted growth (Patriota *et al.* 2023), impaired cognitive development, behavioural problems, and poorer academic performance (Rosen *et al.* 2023). Food insecurity during pregnancy is associated with adverse outcomes, including increased odds of obesity (Nguyen *et al.* 2024), gestational diabetes mellitus, and poorer maternal mental health (Bell *et al.* 2024). While some studies suggest potential implications for infant health, evidence for direct intergenerational impacts remains limited (Bell *et al.* 2024). Food insecurity's impact extends beyond individual health to broader societal issues, perpetuating cycles of poverty by reducing work productivity and increasing healthcare costs for individuals and society (Swinburn *et al.* 2019, Himmelgreen *et al.* 2022). This wide-ranging impact underscores the urgent need for comprehensive policies and interventions to address food poverty and insecurity as a critical public health and social justice issue.

### Policy context in Northern Ireland and Republic of Ireland

The United Nations’ Sustainable Development Goals include eliminating hunger by 2030, acknowledging that food poverty and insecurity are issues faced by countries at all economic levels, including wealthy nations (United Nations General Assembly [UNGA] 2015). Although ROI and NI operate under separate jurisdictions, both regions on the island of Ireland face similar challenges with food poverty and insecurity, exacerbated by factors such as high cost-of-living, low wages, precarious employment, social welfare issues, and housing insecurity (Ipsos and The Trussell Trust 2023, Joseph Rowntree Foundation 2024). The COVID-19 pandemic has further compounded these issues. The ROI was ranked as the most expensive country in the EU for household expenditure on goods and services in 2023, with prices 46% above the EU average (Eurostat 2023). In NI, Brexit and political instability have contributed to rising levels of food poverty and insecurity due to rising food prices as a result of increased bureaucracy adding to the cost of food after Brexit and impacting on lower-income households’ disproportionately higher spend of their income on food than their higher-income counterparts (Barons and Aspinall 2020, Caraher *et al.* 2023).

In response, both governments have primarily supported voluntary and community sector initiatives. For example, the Irish government, through the Fund for European Aid to the most Deprived (FEAD) programme, provides funding to voluntary organizations for food aid distribution (Department of Social Protection 2023). In NI, the government has piloted Social Supermarket initiatives, offering discounted food and support services (Department for Communities 2022). Both jurisdictions have implemented school meal programmes, with the ROI aiming for a universal programme by 2030 and NI providing free school meals based on eligibility criteria (Department of Education 2023). As signatories to the International Covenant on Economic, Social and Cultural Rights, both countries recognize the right to adequate food (Caraher and Furey 2018).

### Food poverty and insecurity in the news media and the importance of framing

With the growing prevalence and policy responses outlined above, understanding how these issues are communicated through news media becomes increasingly important (Wells and Caraher 2014, Kerins *et al.* 2023). News media plays a crucial role in shaping public and political perceptions of social- and health-related issues such as food poverty and insecurity. Framing, as articulated by Entman (1993) in his seminal work in communication studies, involves simplifying complex issues by highlighting specific aspects to make them more salient and memorable to audiences. Drawing from both cognitive psychology and media studies, Entman developed a framework that identifies four key elements in how issues are framed: defining the problem, identifying causes, proposing solutions, and offering moral evaluation. This framework has been widely applied in media analysis to understand how social issues are presented and interpreted, particularly in news coverage of complex social problems. These media frames influence how audiences understand and respond to issues, ultimately informing policy discourse and public response (Matthes 2009).

Recent analyses reveal concerning patterns in how food poverty is framed in news media. A rapid review of media coverage in high-income countries identified a significant disconnect between portrayed causes and solutions: while structural drivers (such as inadequate wages and welfare support) are frequently acknowledged, proposed interventions predominantly focus on charitable responses (Kerins *et al.* 2023). This misalignment potentially undermines effective policy development by directing public attention towards downstream interventions rather than addressing systemic causes (Shiffman and Shawar 2022).

On the island of Ireland, recent studies have highlighted similar challenges in media representation. Analysis of broadcast media found that coverage tends to frame food poverty through narratives of the ‘deserving poor’ and charitable responses (Kerrigan *et al.* 2025), while print media predominantly focuses on food quantity and charitable provision, with limited attention to structural solutions (Vaughan *et al.* 2024). Notably absent across all coverage are the voices of those experiencing food poverty and insights from health and academic experts, resulting in coverage that lacks comprehensive analysis (Kerins *et al.* 2023, Vaughan *et al.* 2024, Kerrigan *et al.* 2025).

While several studies have analysed newspaper coverage of food poverty and insecurity in the UK through content analysis (Wells and Caraher 2014, Knight *et al.* 2018, Price *et al.* 2020, Yau *et al.* 2021), previous research has not captured the perspectives of those involved in creating and shaping media coverage. As evident in our recently published rapid review (Kerins *et al.* 2023), third sector representatives, such as charities and service providers, are central sources that journalists rely on for expert commentary, statistics, and frontline insights into food poverty and insecurity. These organizations often act as intermediaries, amplifying the experiences of those affected while framing the narrative around causes and solutions. While politicians and individuals experiencing food poverty and insecurity also contribute to media discourse, third sector representatives frequently set the agenda and shape how these issues are contextualized and understood in public debate. The key role of third sector representatives in news production processes, and their interplay with media professionals in shaping public understanding of food poverty and insecurity, highlights the importance of examining both perspectives. Recognizing this interrelationship, this study takes a previously unexplored approach by examining the perspectives of both media professionals and third sector service providers through qualitative interviews, aiming to understand how food poverty is understood and communicated.

To develop more effective media communication strategies around food poverty, it is crucial to understand the factors that shape current coverage. This study represents the first comprehensive examination of both news media professionals’ and third sector service providers’ perspectives on food poverty and its media representation. Understanding these perspectives could inform the development of more balanced reporting practices that better align portrayed problems with potential solutions, ultimately contributing to improved public awareness and more effective policy responses.

## MATERIALS AND METHODS

This study was part of a larger project exploring communication of food poverty on the island of Ireland (Vaughan *et al.* 2024). The research adopted a collaborative, interdisciplinary, and integrated knowledge translation approach. A stakeholder advisory panel, including members of the public, media professionals, and food poverty organizations, was embedded in the project.

### Design

Semi-structured qualitative interviews were conducted with news media professionals and third sector service providers. While food poverty and food insecurity are often used interchangeably in the literature as interrelated concepts, this paper primarily uses the term ‘food poverty’ as it is more commonly used in European contexts. During interviews, participants were asked about their preferred terminology when discussing these issues, with their perspectives on language choice presented in the findings. The stakeholder advisory panel advised on recruitment strategies, interview questions, and assisted with dissemination of findings. Ethical approval was obtained from University of Galway (ref: 2023.02.029) and Ulster University (ref: 2023.04.26) Research Ethics Committees.

### Participants

A sampling frame was used to ensure representation of key participants across the ROI and NI. For news media professionals, this included representation from print and broadcast media, and local and national coverage among those reporting on social and health-related issues. The team worked with both the Institution's press office and stakeholder advisory panel to identify potential media participants. The stakeholder advisory panel also helped identify potential participants from third sector organizations addressing food poverty and insecurity through direct service provision, community support, and advocacy work. Participants were invited by email to take part in an interview.

Sample size adequacy was guided by the concept of information power (Malterud *et al.* 2016), considering study aim, participant expertise, and quality of dialogue. Purposive sampling ensured representation of key informants from both media and third sector organizations, enabling rich data from a focused sample. Information power was monitored throughout data collection and analysis, with a sufficient sample achieved when interviews yielded high-quality insights and new data primarily reinforced existing understanding.

### Data collection

All interviews were conducted online via Microsoft Teams between September and December 2023. The interview topic guides were tailored for each participant group (i.e. news media professionals and third sector organization representatives), with input from the stakeholder advisory panel. The guides addressed the following areas: understanding of food poverty—definitions, perceived drivers and solutions, and perceptions of news media representations of food poverty. Following informed consent, interviews were video-recorded and transcribed using Microsoft Teams. Video recordings were deleted after transcription, and transcripts were de-identified to maintain confidentiality.

### Analysis

Using NVivo software for data management, a combination of deductive and inductive coding was performed. First, using Entman’s (1993) framing theory as a framework, transcripts were systematically coded by the first author (C.K.) to identify how participants understood food poverty through three elements: defining the problem (how food poverty was conceptualized), identifying causes (perceived drivers), and proposing solutions (approaches to addressing food poverty). Second, inductive thematic analysis was conducted to examine participants’ perceptions of news media framing of food poverty. This involved systematic coding of all transcripts to identify meaningful patterns in how participants discussed media coverage, grouping similar codes into broader categories, and identifying overarching themes that captured key patterns in the data. The inductive thematic analysis was primarily conducted by the first author (C.K.), with a subset of transcripts independently coded by a second researcher (E.V.). Regular meetings were held between coders to discuss and refine the coding framework, ensuring consistency in code application (Saldana 2021).

After completing the analysis of the full data set, a matrix was then developed to examine patterns across participant groups (media professionals vs third sector representatives) and regions (ROI vs NI). This matrix organized codes by participant group and region, enabling systematic identification of shared perspectives across all participants, areas where views differed between media and third sector representatives, and regional variations in understanding and experiences.

### Researcher positionality

The first author, who conducted all interviews, has a background in clinical dietetics and cardiovascular health charity work. However, they had no prior professional relationships with the participants or their organizations and no direct experience working in the food poverty or media sectors. This lack of direct experience in the studied sectors supported an open, neutral approach during data collection and analysis, reducing the influence of preconceptions on the research process.

## RESULTS

### Participants

The study comprised 30 participants: 16 news media professionals and 14 third sector representatives from the ROI and NI. The interviews averaged 60–75 min for third sector stakeholders and 50–70 min for news media professionals.

Of 72 invited news media professionals, 16 (22%) agreed to take part, with 10 (63%) from the ROI and 6 (37%) from NI. Participants emanating from the news media represented diverse media types: newspapers [including both broadsheet and tabloid formats (although categorization was not always clear-cut due to differences between online and print versions)] (25%, *n* = 4), radio [across public (*n* = 1) and independent broadcasting (*n* = 3)] (25%, *n* = 4), television (19%, *n* = 3), and combined print and broadcast media (31%, *n* = 5). Coverage was equally split between national (50%, *n* = 8) and regional/local (50%, *n* = 8). Roles included news producers and editors (*n* = 5), journalists (*n* = 6), and broadcasters (*n* = 5).

From 19 invited third sector organizations, 14 (74%) participated, with 8 (57%) based in the ROI and 6 (43%) in NI. These organizations represented a range of services addressing food poverty, including food banks, community support centres, and advocacy groups. Participants occupied various positions within these organizations: CEOs and Founders of charity organizations (*n* = 3), senior management and staff (*n* = 7), research and policy officers (*n* = 3), and a social care worker (*n* = 1).

### Contextual understanding of food poverty and insecurity

This section explores how news media professionals and third sector stakeholders conceptualize food poverty and insecurity, their perceptions of the drivers behind these issues, and the solutions they propose.

#### Definition and understanding of food poverty and insecurity

News media professionals and third sector stakeholders described food poverty and insecurity primarily in terms of insufficient food quantity and poor dietary quality. Third sector stakeholders focused on difficulties in accessing adequate food for families, while news media professionals often linked the issue to consumption of ultra-processed foods and lack of home-cooked meals. Some media professionals noted that a broader group of people, influenced by their upbringing and food environment, may consume processed and cheap foods without necessarily identifying as experiencing food poverty.

*… there's a far bigger group of people because of how they’ve been brought up, they buy a lot of processed foods, a lot of cheap foods that they don't necessarily see themselves as being in food poverty* (Local Radio Broadcaster, ROI).

In addition to these broader societal patterns, some media professionals also highlighted how food poverty can lead to unequal food distribution within households, with parents, particularly mothers, sacrificing their own nutrition to ensure children have enough to eat.

*… in a household, while the children may be eating, that the mum necessarily isn’t… not enough to go around* (Freelance Journalist for Print & Broadcast Media, NI).

Many third sector stakeholders and media professionals expressed a preference for the term ‘food insecurity’ over ‘food poverty’, perceiving it as less stigmatizing, more nuanced, inclusive of those at risk or on the margins, and better representing the interconnected nature of problems within the food system.

*I know that people don’t like that term [food poverty]. I don’t know if it’s because there’s a stigma attached to it and food insecurity sounds, I don’t know if it sounds a bit more pleasant… I suppose language is important* (Supervisor for Charity, NI).

However, some media professionals in NI highlighted ‘poverty’ was their preferred term or language of choice over food poverty, when referring to individuals or families unable to afford basic foods items:

*I’m familiar with all of those terms, food poverty and food insecurity and so on… I would kind of just view that as poverty, you know, if you can’t afford the basics, that’s the problem* (Freelance Journalist for Print & Broadcast Media, NI).

Mental health consequences were a primary concern across stakeholders. Third sector representatives also emphasized physical health effects and impacts on child development, growth, and educational attainment. Additionally, they highlighted social dimensions, including cultural appropriateness and social exclusion.

*People who don’t ever get the chance to sit down around the table with others for a meal, there’s some degree of food poverty in that* (Founder of Charity, NI).

Some media professionals reported on crime as both a consequence and cause of food poverty, including shoplifting and involvement of organized crime gangs.

*… because of the particular nature of northern society and there’s an element of a paramilitary involvement whenever it comes to poverty… There have been some horrendous stories… women who’ve had to borrow money to pay a bill, and then they end up having to… pay back 10 times over or have been forced into prostitution* (Freelance Journalist for Print & Broadcast Media, NI).

*… they’re shoplifting to put food on the table* (Local Radio Broadcaster, ROI).

#### Perceived drivers of food poverty and insecurity

News media professionals and third sector stakeholders in NI emphasized political factors as key drivers of food poverty and insecurity. Primary concerns included the Universal Credit system (the UK's main means-tested social security payment for working-age people)—including the 5-week wait for initial payments, benefit system changes, and removal of the pandemic uplift—and the impact of political instability.

*The process of re-evaluating people with any kind of disability or medical issues was brutal here over the last five or six years where, it was almost everybody got denied and had to appeal. And so many got overturned on appeal, but like the unnecessary length of delay in [social security] payment…* (CEO of Charity, NI).

In contrast, stakeholders in the ROI, particularly media professionals, placed greater emphasis on individual factors. They particularly highlighted levels of education and knowledge linked to cooking and understanding of healthy eating as key drivers.

*… people living in disadvantaged areas… not understanding or knowing… eating things to keep their children full straight away as opposed to what the healthy aspects are* (Local Radio Broadcaster, ROI).

However, third sector stakeholders identified that structural barriers, particularly inadequate facilities in emergency accommodation, often prevented people from cooking despite having the knowledge and desire to do so.

*One of the key concerns that we would have is for families living in direct provision or families living in emergency accommodation… they do not have access to cooking facilities…* (Senior Research & Policy Manager of Charity, ROI).

Across both regions, the impact of the COVID-19 pandemic and resulting economic fallout was consistently cited as a significant driver of food poverty and insecurity, often bringing new demographics into contact with support services for the first time.

*… COVID came along and that kind of increased and it opened up avenues where there were so many other sort of groups of people who were falling through the gaps. And from COVID really, we haven't really looked back and that trajectory of people who find themselves living in real hardship and real difficulty continues to go upward* (Founder of Charity, NI).

#### Perceived solutions to address food poverty and insecurity

Stakeholders across both regions acknowledged the prominence of community and voluntary sector responses to food poverty and insecurity, while recognizing the limitations of these approaches and the need for more comprehensive solutions. Third sector stakeholders reported an evolution in their approach, moving towards more holistic support and sustainable community action.

*… it’s not just sort of handing over a few parcels of food for families……we now have a wrap-around service where people can get advice around housing, mortgage payments, debt management, and we point people towards training and employment* (Founder of Charity, NI).

Government actions were viewed differently across regions. In the ROI, stakeholders noted positive actions, particularly the expansion of the school meals programme, while emphasizing the need for further improvements. In NI, where the government was not functioning at the time of data collection, stakeholders emphasized the need for political restoration and subsequent policy implementation.

*… the Minister’s commitment…for universal provision of hot school meals…they are not only on track but really moving ahead in terms of that level of investment* (Senior Research & Policy Manager of Charity, ROI).

*… it’s calling for the restoration of the Executive, calling for the full implementation of the Anti-Poverty Strategy and its recommendations and calling for a Childcare Strategy to be fully implemented as soon as possible when the Government is back up and running…* (Strategic Development Manager of Charity, NI).

Across both regions, stakeholders called for welfare reform, including benchmarking social welfare payments to living costs. However, they also emphasized the multifaceted nature of food poverty and insecurity, acknowledging that addressing these issues requires a complex, multi-pronged approach.

*… there’s no silver bullet for the issues that people are facing in terms of food poverty, but it’s a cocktail of just disaster that requires action at multiple levels* (Editor of Regional Newspaper, NI).

### Stakeholder perceptions of news media framing of food poverty and insecurity

This section examines how news media professionals and third sector stakeholders perceive the media coverage of food poverty and insecurity. Table 1 presents the key findings from the inductive analysis of participants’ perceptions, organized by theme and category, and indicates whether each code was discussed by media professionals, third sector representatives, or both.

**Table 1.** Themes, categories, and codes identified through inductive analysis of participants’ perceptions of news media framing of food poverty.

Theme	Category	Codes
Media coverage focus and drivers	Reactive coverage	Event-driven (MP, TS), Seasonal fluctuations (MP)
	Simplistic framing	Lack of nuance (TS), Complex issue (MP, TS)
Representation in reporting	Preferred case studies	Families (MP), Women and children (MP), Impactful stories (MP)
	Challenges securing case studies	Stigma (MP), Ethical concerns (TS)
Interdependence of media and third sector	Mutual reliance	Securing case studies (MP), Information sharing (MP)
	Problematic dynamics	Decontextualized data (TS), PR driven by fundraising (MP)
Challenges in reporting	Resource constraints	Time and resources (MP), Changing revenue models (MP)
	Engaging the public	Complexity of issue (MP, TS), Balancing depth and interest (MP)
	Gaps in reporting	Limitations of print media (MP), Data and expertise (MP)

*Note*. Based on inductive analysis of full data set (*n* = 30; 16 media professionals and 14 third sector representatives).

MP, media professionals; TS, third sector representatives.

#### Media coverage focus and drivers

News media professionals reported that their coverage of food poverty and insecurity predominantly centred on food banks and other charitable initiatives. This focus was reactive, often driven by the release of reports or figures from third sector organizations, as well as spikes in demand for their services.

*… we would be talking about there being, for example, 150% increase in the number of people using the food banks… figures like these released [by charities]* (Journalist for Regional Newspaper, NI).

This event-driven reporting contributed to fluctuations in coverage throughout the year, with increased attention during holiday periods and in response to economic challenges such as the cost-of-living crisis.

*… it’s very focused around Christmas time… there’s this enormous focus on food at Christmas time here in this country… what happens in January, February, there’s still people actually who are at risk of food poverty, and you don’t hear a flipping word about them in the media, only at Christmas* (Manager with Charity, ROI).

Some third sector stakeholders expressed concerns that this approach to coverage often resulted in a simplistic view of food poverty and insecurity, lacking nuance and context.

*We’re probably a good few years away from having media coverage that’s like more wanting to talk more about like the general nuances and how it interweaves in and out of like different families and different groups* (National Policy Manager for Charity, ROI).

Both groups highlighted the complex nature of food poverty and insecurity as a factor contributing to the narrow scope of coverage. The absence of clear solutions or call to action was cited as a particular challenge.

*I think it's very complicated and the media in general doesn't like complicated stories… no-one knows what the solution really is…* (Local Radio Broadcaster, ROI).

*… there is no crisp and clean and easy answer to any of the stuff that we’re facing. And so, the complexity of the question is boring to the general public and so it's not going to sell papers* (CEO of Charity, NI).

#### Representation in reporting

News media professionals expressed a preference for stories focusing on families, women, and children experiencing food poverty and insecurity, considering them more impactful for engaging readers and listeners.

*Most headlines… usually talk about how parents make decisions to try and feed their children well… Editors tend to look at stories with children as more impactful* (Editor of National Newspaper, ROI).

Some media professionals in the ROI highlighted how personal stories can help destigmatize food poverty and encourage others to come forward.

*So, the first thing you want to hear is the story of a person who is experiencing this, because that's the best way people can relate, and it's also the best way people feel they can come forward themselves and destigmatise it* (Correspondent with National Broadcaster, ROI).

However, news media professionals highlighted significant challenges in securing participation from individuals experiencing food poverty and insecurity, primarily due to the stigma.

*No one wants to go on TV or be pictured in a newspaper saying like I’m experiencing food poverty…the stigma around it* (Broadcast Producer, ROI).

Third sector stakeholders expressed discomfort in their role as gatekeepers for media access to individuals experiencing food poverty and insecurity, highlighting ethical concerns.

*I don't want to cross the line or boundary or ask them because I don't want to put them on the spot* (Social Care Worker with Charity, ROI).

Despite these challenges, news media professionals emphasized the importance of case studies for public engagement and potential policy influence.

*… if you get powerful case studies or really strong pictures that actually resonate with the viewer and that they remember and then that they can actually…prompt action from those in power* (Broadcast Producer, ROI).

Some media professionals highlighted that the lack of case studies in news coverage can impact public perception and understanding of food poverty by failing to provide a comprehensive picture of the diverse experiences and circumstances of those affected.

*When you talk about the general public's idea…there is a kind of a misconception of what a particular group [those affected by food poverty] is, which is exacerbated by not having case studies, people talking about who they are and what they do* (National News Reporter, ROI).

To address these challenges, some media professionals suggested the importance of advance planning and collaboration with charities.

*It’s about planning…say for example [charity name] know they have a survey coming out next week or in the next few months… They know that the first question which will be asked is have you a case study… So, it requires so much planning to identify that person willing to speak…* (Correspondent with National Broadcaster, ROI).

#### Interdependence of media and third sector

Both news media professionals and third sector stakeholders acknowledged a mutual reliance in reporting on food poverty and insecurity. This interdependence was particularly evident in the processes of securing case studies and sharing information.

*It’s also about making sure that we’re fed with the information so that we know what’s happening on the ground at the moment as I haven’t a clue…* (Correspondent with National Broadcaster, ROI).

Some stakeholders highlighted that the voluntary sector was feeding into surface-level coverage of food poverty by providing decontextualized data, which the media accept at face value:

*… voluntary sector organisations or other organisations release reports with headline stats with very little information behind those stats and the media don’t question it and query it… The media sometimes want to be -- spoon-fed is probably a derogatory term -- but they want it broken down… and they don’t take the time to read the longer reports and really delve into it* (National Policy Manager of Charity, ROI).

Some news media professionals highlighted that the voluntary sector's approach to public relations was often led by fundraising needs rather than addressing systemic issues.

*Suppose like most of them [charities], are staffed by good people with very solid and noble intentions but they do get into rhythm of being led by the publicity, like led by fundraising* (Editor of National Newspaper, ROI).

#### Challenges in reporting

News media professionals reported significant challenges in reporting on food poverty and insecurity, including time and resource constraints. These pressures were further exacerbated by changing news media revenue models, which affected the overall capacity for comprehensive coverage.

*There’s definitely time pressures, just resource pressures. It’s so expensive to do investigations and it’s so time intensive and it’s so cash expensive… we have a crisis in journalism, that advertising is not where it was before the pandemic… So, the model is different and then the revenue model is different because people don’t pay for their news anymore.* (Editor of National Newspaper, ROI).

Both groups noted difficulties in engaging the public with complex issues, balancing content depth with perceived public interest, and finding new angles and perspectives when reporting on persistent issues like food poverty. News media professionals reported that readers are often more inclined towards reading about the negative aspects of an issue rather than potential solutions.

*… trying to find a way of engaging the viewer in what is essentially the same story over and over and over again* (Broadcast Producer, NI).

Despite these challenges, some news media professionals acknowledged a responsibility to inform the public about important issues, although the tension between this responsibility and the business considerations of private media companies was highlighted.

*But we do take it quite seriously that we have duties… to inform them about things they need to know about, like there's always a bit of tension between what will sell papers and what people need to know* (Editor of National Newspaper, ROI).

The limitations of print media, particularly in terms of article word limits, for in-depth coverage were also highlighted. Broadcast media, such as radio and television, were seen as more conducive to in-depth discussions on the government's role in addressing food poverty.

*… in terms of radio and TV [discussions on government role in food poverty] freely take off whereas like someone might read it in the newspaper, oh yeah, like, you know, the charity's saying XYZ, but like, it's only when you hear that maybe in broadcast that you like a real understanding of it… It's extremely difficult trying, I suppose, tell that story in a newspaper in 500 words* (Broadcast Producer, ROI).

#### Data and expertise needs

News media professionals highlighted significant gaps in data and expertise that affected the quality and depth of reporting on food poverty and insecurity. In the ROI, some news media professionals emphasized the need for better, independent data, expressing concerns about reliance on charity-provided information.

*We need centralised data and more independent… Like as a centralised CSO [Central Statistics Office] thing… we do need a more independent look at exactly what’s happening… I’m not saying we wouldn’t trust charities or that they don’t do a good job. It’s just that, yeah, it’s their story then and not the story that we would put together* (Editor of National Newspaper, ROI).

In NI, some media professionals noted the absence of academic voices in the coverage of food poverty, suggesting that this may be due to journalists’ lack of awareness about relevant experts. They contrasted this with other areas, such as healthcare, where journalists regularly engage with academics for analysis and solutions.

*So, it would be really useful to find out who, who the academics are, and who would want to speak out… To ask them what needs to be done and tell us the solutions* (Broadcast Producer, NI).

## DISCUSSION

The overall aim of this study was to explore how news media professionals and third sector stakeholders understand and communicate the topic of food poverty. Among all participants, distinct perspectives were apparent as to the perceived causes of and potential solutions to the problem, and these occurred largely between the two groups and across discrete ideological lines. Most of those working in third sector organizations tended to emphasize the structural drivers of food poverty and therefore advocated policy solutions. Views on drivers and solutions among media professionals, however, were more mixed. While many acknowledged the upstream drivers of the problem, more participants in this cohort expressed the view that food poverty was an individual issue, driven by lifestyle, or lack of knowledge or skills. These competing and overlapping discursive frames are also reflected in media coverage of food poverty in Ireland, where there is frequently an emphasis on upstream drivers, but an outsized and discordant focus on downstream charitable solutions (Kerrigan *et al.* 2025). In this context, in this section we highlight for discussion some of the key findings, focusing specifically on the risks of individualizing food poverty.

### The risk of individualizing food poverty

While evidence points to the structural nature of food poverty (Dowler and O’Connor 2012), our analysis suggests that institutional arrangements between media and charitable organizations may inadvertently reinforce individualistic narratives. This reflects broader patterns identified in poverty reporting elsewhere (McKendrick *et al.* 2008, Moore 2020), where tensions arise between media needs for immediate, newsworthy stories, and third sector focus on advocacy and protecting service users. A key finding of this study was that the use of individual case studies, with preference for single mothers and children, was the preferred lens through which media professionals tell the story of food poverty. Such case studies—whereby journalists rely on the participation of affected individuals to personalize and embody the issue—were viewed by journalists as more impactful. It is unclear, however, if this is the case. A classic experiment by Iyengar (1996) found that with episodic framing of poverty, whereby there is a specific focus on individual victims rather than a thematic or general focus, viewers were more likely to assign greater responsibility for poverty to the individual, and that the effect was greatest where reporting was gendered and single mothers were depicted. Subsequent experimental work further suggests that while episodic reporting can trigger an effective response that elicits sympathy for the individual, this does not necessarily translate to support for policy change (Gross 2008). In part, this may be because people tend to believe there is greater social mobility than there actually is and thus individualistic explanations for poverty appeal to pre-existing biases (Davis and Williams 2020). Regardless of the mechanisms at work, at the very least there is a risk that case studies may detract from the wider structural underpinnings of food poverty by personalizing rather than politicizing the issue.

The preference for individual case studies in media reporting on food poverty not only risks detracting from structural causes but also reflects a broader tension in how the issue is conceptualized and communicated. This individualization is further reflected in terminological preferences. While many news media and third sector stakeholders expressed a preference for ‘food insecurity’ over ‘food poverty’, perceiving it as less stigmatizing, some media professionals preferred to frame these issues directly as ‘poverty’. This perspective aligns with debates in the literature regarding framing of this issue. For example, Lambie-Mumford *et al.* (2014) positions food insecurity as a symptom of broader structural issues including poverty and economic pressures; Dowler and O’Connor (2012) connect food access issues to the right to food, requiring poverty to be addressed as the root cause; and Van der Berg *et al.* (2022) frame food insecurity as a branch of poverty requiring policy-level intervention. This terminological tension mirrors the disconnect between acknowledged structural causes and individualized solutions observed in media coverage, with important implications for how issues are understood and addressed in public discourse.

### Media representation, stigma power, and responsibilization

Insofar as the impact of case studies may be over-stated, the harm they may cause may possibly be under-estimated. Aside from the obvious ethical implications highlighted by participants in obtaining case studies (Moore 2020), additional issues relate to the politics of representation, and the potential stigmatizing impact on individuals experiencing poverty. Symbolic-structural frameworks for conceptualizing stigma and discrimination have highlighted how stigma is operationalized by leveraging symbolic violence through discursive and cultural outputs to normalize and legitimize social inequality (Parker and Aggleton 2003). A rapid review of studies (*n* = 22) exploring individuals’ experiences of poverty stigma and health and well-being reported that participants across multiple studies in different countries believed that the media promoted negative stereotypes of those on lower incomes and people living in poverty (Inglis *et al.* 2023). Stigma scholars have suggested that such representations serve a purpose, operating as a form of stigma power (Link and Phelan 2014, Tyler 2020) to simultaneously shore up politics and policies that embed poverty by design, and keep those experiencing poverty ‘down, in or away’ (Link and Phelan 2014). Tyler (2015, 2020), for instance, points to the cultural and political discourse that emerged during the austerity era, which frequently positioned recipients of welfare as the antithesis to the model self-sufficient neo-liberal subject, while responsibilizing them for their own experiences of precarity (Butler 2015). Similarly, while some media professionals in this study perceived food poverty organizations as insufficiently focused on policy advocacy, such advocacy may be beyond their remit and capacity and represents a further example of downstream responsibilization of individual and community actors rather than holding those in power to account for the social and structural deficits actually driving the inequalities that lead to poverty in the first place.

### Implications for health promotion practice

The findings of this study have important implications for health promotion practice. First, they highlight the need for researchers and health promotion practitioners to work strategically and collaboratively with both media professionals and third sector organizations to reframe the narrative around food poverty. Such reframing is essential given how issues are represented in public discourse shapes both policy responses and public support for structural interventions (Matthes 2009, Shiffman and Shawar 2022). In the UK, one such collaboration between the Joseph Rowntree Foundation and Frameworks UK (2025) has led to significant changes to the way activists and spokespersons in the former organization communicate about poverty, with the aim of shifting the narrative, reducing stigma and changing public opinion. Similarly, there are opportunities for third sector organizations to re-imagine how they operate. For example, in NI, community organization FoodStock has positioned itself not as a charity or food bank, but rather as a social solidarity initiative that emphasizes the rights and inherent dignity of all the people they work, while also highlighting gaps in public provision of the community's needs.

Second, there is a role for health promotion in building capacity among journalists and third sector organizations to better communicate the structural determinants of food poverty. Comparable initiatives have proven effective in improving reporting on suicide and destigmatizing mental health issues; for example, SHINE is a third sector organization that supports people with experience of ill mental health in Ireland. Among their core missions is the de-stigmatization of mental ill health. As part of that mission, one of their flagship programmes is Headline (2025), which is specifically aimed at working collaboratively with media professionals to improve reporting on mental health and suicide. Initiatives focused on meeting this aim include a journalism fellowship, a mental health reporting awards programme, and a variety of training and educational resources for those working in the media.

Finally, the study underscores the importance of moving beyond charitable responses to food poverty towards more sustainable, systemic solutions. While charitable responses may provide immediate relief, they risk normalizing food poverty and obscuring the state's responsibility to protect citizens’ right to food. Health promotion practitioners should advocate for and support the development of evidence-based policy solutions that address the structural drivers of food poverty, while working to build public and political support for such measures through effective communication strategies.

### Limitations and future research directions

This study has several limitations. First, the low response rate among media professionals (22%) suggests potential selection bias, as findings may primarily reflect the views of those most willing to engage with the topic. This should be considered when interpreting the results. Additionally, while the study included both media and third sector perspectives, it did not directly incorporate the voices of those with lived experiences of food poverty. Integrating these perspectives in future research could provide a more comprehensive understanding of food poverty representation. Furthermore, the study's focus on the island of Ireland provides an in-depth exploration of food poverty representation within a specific socio-political context, which may limit generalizability to other settings. The findings also represent a snapshot of stakeholder perspectives at a single point in time, while food poverty and its media representation are continually evolving. Future research could benefit from cross-national comparisons and longitudinal designs to provide a more comprehensive understanding of food poverty framing across contexts and over time.

## CONCLUSION

This study provides novel insights into how food poverty is understood and communicated by media professionals and third sector organizations on the island of Ireland. The findings reveal how embedded journalistic conventions and institutional arrangements between news media and charitable organizations may inadvertently reinforce individualistic narratives, despite evidence supporting structural causes of food poverty. This suggests the need for strategic approaches to support more effective communication to emphasize the human rights issue and facilitate access to independent data sources and academic expertise, while being mindful of ethical considerations in representation. This involves developing best practice guidelines for ethical reporting of food poverty, along with building capacity among both journalists and third sector organizations to better communicate structural determinants, to help create more balanced coverage that supports effective policy responses.

## Data Availability

The data underlying this article cannot be shared publicly in order to protect the privacy of individuals participating in the study.

## References

[daaf073-B1] Barons MJ, Aspinall W. Anticipated impacts of Brexit scenarios on UK food prices and implications for policies on poverty and health: a structured expert judgement approach. BMJ Open 2020;10:e032376. 10.1136/bmjopen-2019-032376 PMC7059525 32132136

[daaf073-B2] Bell Z, Nguyen G, Andreae G et al Associations between food insecurity in high-income countries and pregnancy outcomes: a systematic review and meta-analysis. PLoS Med 2024;21:e1004450. 10.1371/journal.pmed.1004450 39255262 PMC11386426

[daaf073-B3] Butler J . Notes Toward a Performative Theory of Assembly. Cambridge, MA: Harvard University Press, 2015.

[daaf073-B4] Caraher M, Furey S. The Economics of Emergency Food aid Provision: A Financial, Social and Cultural Perspective. London: Palgrave Macmillan, 2018.

[daaf073-B5] Caraher M, Furey S, Wells R. Food Policy in the United Kingdom: An Introduction. London: Routledge, 2023.

[daaf073-B6] Davis O, Geiger BB. Did food insecurity rise across Europe after the 2008 crisis? An analysis across welfare regimes. Soc Policy Soc 2017;16:343–60. 10.1017/S1474746416000166

[daaf073-B7] Davis RP, Williams WR. Bringing psychologists to the fight against deep poverty. Am Psychol 2020;75:655–67. 10.1037/amp0000650 32673009

[daaf073-B8] Department for Communities . Social Supermarket Support Screening. Northern Ireland: Department for Communities, 2022.

[daaf073-B9] Department of Education . School Meals Programme Review. Ireland: Department of Education, 2023.

[daaf073-B10] Department of Social Protection . FEAD Programme Ireland Annual Implementation Report 2023. Ireland: Department of Social Protection, 2023.

[daaf073-B11] Dowler EA, O’Connor D. Rights-based approaches to addressing food poverty and food insecurity in Ireland and UK. Social Sci Med 2012;74:44–51. 10.1016/j.socscimed.2011.08.036 22000764

[daaf073-B12] Entman RM . Framing: toward clarification of a fractured paradigm. J Commun 1993;43:51–8. 10.1111/j.1460-2466.1993.tb01304.x

[daaf073-B13] Eurostat . Comparative Price Levels of Consumer Goods and Services in the European Union. Luxembourg: Eurostat, 2023.

[daaf073-B14] FAO, IFAD, UNICEF, WFP, and WHO . The State of Food Security and Nutrition in the World 2023. Rome: FAO, IFAD, UNICEF, WFP, and WHO, 2023.

[daaf073-B15] Food Poverty Working Group . Food Poverty: Government Programmes, Schemes and Supports. Ireland: Department of Social Protection, 2022.

[daaf073-B16] Food Standards Agency . Food and You 2: Wave 8. United Kingdom: Food Standards Agency, 2024.

[daaf073-B17] FrameWorks UK . (2025) *Talking about poverty*. https://frameworksuk.org/changing-the-story/talking-about-poverty/ (28 March 2025, date last accessed).

[daaf073-B18] Gross K . Framing persuasive appeals: episodic and thematic framing, emotional response, and policy opinion. Polit Psychol 2008;29:169–92. 10.1111/j.1467-9221.2008.00622.x

[daaf073-B19] Gundersen C, Ziliak JP. Food insecurity research in the United States: where we have been and where we need to go. Appl Econ Perspect Policy 2018;40:119–35. 10.1093/aepp/ppx058

[daaf073-B20] Headline . (2025) *About Headline*. https://headline.ie/ (28 March 2025, date last accessed).

[daaf073-B21] Himmelgreen DA, Romero-Daza N, McCord C. Using syndemic theory to understand food insecurity and diet-related chronic diseases. Soc Sci Med 2022;292:114619. 10.1016/j.socscimed.2021.114619 32586635

[daaf073-B22] Inglis G, Jenkins P, McHardy F et al Poverty stigma, mental health, and well-being: a rapid review and synthesis of quantitative and qualitative research. J Community Appl Soc Psychol 2023;33:783–806. 10.1002/casp.2677

[daaf073-B23] Ipsos and The Trussell Trust . Hunger in Northern Ireland. Northern Ireland: The Trussell Trust, 2023.

[daaf073-B24] Iyengar S . Framing responsibility for political issues. Ann Am Acad Pol Soc Sci 1996;546:59–70. 10.1177/0002716296546001006

[daaf073-B25] Joseph Rowntree Foundation . UK Poverty 2024: The Essential Guide to Understanding Poverty in the UK. York: Joseph Rowntree Foundation, 2024.

[daaf073-B26] Kantilafti M, Giannakou K, Chrysostomou S. Multimorbidity and food insecurity in adults: a systematic review and meta-analysis. PLoS ONE 2023;18:e0288063. 10.1371/journal.pone.0288063 37410753 PMC10325088

[daaf073-B27] Kerins C, Furey S, Kerrigan P et al News media framing of food poverty and insecurity in high-income countries: a rapid review. Health Promot Int 2023;38:daad188. 10.1093/heapro/daad188 38150220 PMC10752350

[daaf073-B28] Kerrigan P, McCartan A, Kerins C et al Between headlines and hunger: media framing of food poverty on the Island of Ireland. Media Cult Soc 2025;47:680–98. 10.1177/01634437241299342

[daaf073-B29] Knight A, Brannen J, O'Connell R et al How do children and their families experience food poverty according to UK newspaper media 2006–15? J Poverty Social Justice 2018;26:207–23. 10.1332/175982718X15200701225223

[daaf073-B30] Lambie-Mumford H, Crossley D, Jensen E et al Household Food Security in the UK: A Review of Food Aid. London: Department for Environment, Food and Rural Affairs, 2014.

[daaf073-B31] Link BG, Phelan J. Stigma power. Soc Sci Med 2014;103:24–32. 10.1016/j.socscimed.2013.07.035 24507908 PMC4451051

[daaf073-B32] Loopstra L . Interventions to improve nutrition in urban areas interventions to address household food insecurity in high-income countries. Proc Nutr Soc 2018;77:270–81. 10.1017/S002966511800006X 29580316

[daaf073-B33] Lopes SO, Abrantes LCS, Azevedo AFM et al Food insecurity and micronutrient deficiency in adults: a systematic review and meta-analysis. Nutrients 2023;15:1074–14. 10.3390/nu15051074 36904074 PMC10005365

[daaf073-B34] Malterud K, Siersma VD, Guassora AD. Sample size in qualitative interview studies: guided by information power. Qual Health Res 2016;26:1753–60. 10.1177/1049732315617444 26613970

[daaf073-B35] Matthes J . What’s in a frame? A content analysis of media framing studies in the world’s leading communication journals, 1990–2005. Journal Mass Commun Q 2009;86:349–67. 10.1177/107769900908600206

[daaf073-B36] McKendrick JH, Sinclair S, Irwin A et al The Media, Poverty and Public Opinion in the UK. Joseph Rowntree Foundation, 2008.

[daaf073-B37] Middleton G, Mehta K, McNaughton D et al The experiences and perceptions of food banks amongst users in high-income countries: an international scoping review. Appetite 2018;120:698–708. 10.1016/j.appet.2017.10.029 29079476

[daaf073-B38] Moore K . Reporting on Poverty: News Media Narratives and Third Sector Communications in Wales. Cardif: Cardiff University Press, 2020.

[daaf073-B39] Nguyen G, Bell Z, Andreae G et al Food insecurity during pregnancy in high-income countries, and maternal weight and diet: a systematic review and meta-analysis. Obes Rev 2024;25:e13753. 10.1111/obr.13753 38693587

[daaf073-B40] Northern Ireland Statistics and Research Agency . Family Resources Survey: Northern Ireland 2022/23. Northern Ireland: NISRA, 2024.

[daaf073-B41] O’Connor N, Farag K, Baines R. What is food poverty? A conceptual framework. Br Food J 2016;118:429–49. 10.1108/BFJ-06-2015-0222

[daaf073-B42] Parker R, Aggleton P. HIV and AIDS-related stigma and discrimination: a conceptual framework and implications for action. Soc Sci Med 2003;57:13–24. 10.1016/S0277-9536(02)00304-0 12753813

[daaf073-B43] Patriota PF, Silva A, Neri DA et al Association between household food insecurity and stunting in children aged 0–59 months: systematic review and meta-analysis of cohort studies. Matern Child Nutr 2023;19:e13438. 10.1111/mcn.13438 38196291 PMC10981479

[daaf073-B44] Pourmotabbed A, Moradi S, Babaei A et al Food insecurity and mental health: a systematic review and meta-analysis. Public Health Nutr 2020;23:1778–90. 10.1017/S136898001900435X 32174292 PMC10200655

[daaf073-B45] Price C, Barons M, Garthwaite K et al ‘The do-gooders and scroungers’: examining narratives of foodbank use in online local press coverage in the West Midlands, UK. J Poverty Social Justice 2020;28:279–98. 10.1332/175982720X15905998323834

[daaf073-B46] Rosen JG, Vazquez D, Simonyan D et al Associations between food insecurity and child and parental physical, nutritional, psychosocial and economic well-being globally during the first 1000 days: a scoping review. Matern Child Nutr 2023;19:e13421. 10.1111/mcn.13574 PMC10750018 37828823

[daaf073-B47] Saldana J . The Coding Manual for Qualitative Researchers. 4th edn. Thousand Oaks, CA: Sage Publications, 2021.

[daaf073-B48] Shiffman J, Shawar YR. Strengthening accountability of the global health agenda: the role of frame conflicts. Global Health 2022;18:1–8. 10.1186/s12992-021-00793-2 34980187 PMC8725488

[daaf073-B49] Swinburn BA, Kraak VI, Allender S et al The global syndemic of obesity, undernutrition, and climate change: the Lancet Commission report. The Lancet 2019;393:791–846. 10.1016/S0140-6736(18)32822-8 30700377

[daaf073-B50] Tarasuk V, Mitchell A. Household Food Insecurity in Canada, 2017–18. Research to Identify Policy Options to Reduce Food Insecurity (PROOF). Toronto: University of Toronto, 2020.

[daaf073-B51] Tyler I . Classificatory struggles: class, culture and inequality in neoliberal times. Sociol Rev 2015;63:493–511. 10.1111/1467-954X.12296

[daaf073-B52] Tyler I . Stigma: The Machinery of Inequality. London: Zed Books, 2020.

[daaf073-B53] United Nations General Assembly . Transforming Our World: The 2030 Agenda for Sustainable Development. New York: United Nations, 2015.

[daaf073-B54] Van der Berg S, Patel L, Bridgman G. Food insecurity in South Africa: evidence from NIDS-CRAM wave 5. Dev South Afr 2022;39:722–37. 10.1080/0376835X.2022.2062299

[daaf073-B55] Vaughan E, Kerins C, Furey S et al Communicating Food Poverty on the Island of Ireland. Cork: Safefood, 2024.

[daaf073-B57] Wells R, Caraher M. UK print media coverage of the food bank phenomenon: from food welfare to food charity? Br Food J 2014;116:1426–45. 10.1108/BFJ-03-2014-0123

[daaf073-B58] Yau A, Singh-Lalli H, Forde H et al Newspaper coverage of food insecurity in UK, 2016–2019: a multi-method analysis. BMC Public Health 2021;21:1201. 10.1186/s12889-021-11214-9 34238270 PMC8268386

